# Ion chamber measurements of transverse gamma knife beam profiles

**DOI:** 10.1120/jacmp.v3i1.2587

**Published:** 2002-01-01

**Authors:** Morris I. Bank

**Affiliations:** ^1^ Indiana University Medical Center Radiation Oncology 535 Barnhill Drive Indianapolis Indiana 46202

**Keywords:** gamma knife, radiation therapy, beam profiles

## Abstract

A microchamber, PTW Pinpoint 31006, was used to measure transverse beam profiles for an Elekta Gamma Knife, Model B, and compared with profiles measured with film dosimetry. The microchamber sensitive volume has a diameter of 2 mm, which is smaller than the gamma knife beams, and a length of 5 mm. The chamber was mounted in a custom cassette in a spherical plastic phantom, supplied by Elekta, and oriented in a sagittal plane with the 2‐mm dimension at right angles to the transverse plane. The phantom was manually moved across the beam, using the gamma knife *x*‐coordinate trunnions, to measure the profiles. Profiles were also measured with V‐film placed in a cassette mounted in the spherical plastic phantom. The films were scanned with a Scanditronix film scanner and converted to dose with a density to dose calibration curve. The results were superimposed for comparison. The beam width at the 50% intensity was measured from the film profiles to give the dimensions of the beams in the orthagonal planes. The ion chamber measurements are compared with the film results for the transverse, *x* profiles. Good agreement between the film and ion chamber transverse profiles is observed.

PACS number(s): 87.66.–a, 87.53.–j, 87.53.Ly

## INTRODUCTION

The Elekta Leksell Gamma Knife has 201 Co‐60 sources to form the clinical treatment volume. The 201 sources are positioned on the surface of a hemisphere with the radiation beams directed towards the isocenter of the hemisphere. Primary collimators, fixed in the main body of the gamma knife, shape the primary beam, while interchangeable helmets provide secondary collimation to shape the final treatment beam. Each helmet has 201 individual secondary collimators mated to each of the primary collimators and the associated 201 Co‐60 sources. Four helmets with different size collimators are available to form the clinical beam utilizing the 201 individual beams with nominal clinical beam sizes of 4, 8, 14, and 18 mm. The clinical beam is actually a spherical volume and these nominal sizes refer to the width of the 50% diameter.

Profiles of the composite gamma knife beams are measured to determine the size of the delivered dose distribution. Film is used to measure beam characteristics including profiles from which isodose distributions are determined.^1^ ^,^ ^2^ Film has been used due to lack of a suitable ion chamber with small dimensions relative to the clinical gamma knife beam size, and problems in orienting an ion chamber in the gamma knife beam. Ion chamber measurements are normally considered the “gold standard” for radiation beam measurements due to inherent problems with film dosimetry, such as energy dependence and nonlinearity of response.^3^ ^,^ ^4^ Recently a new ion chamber with a 2‐mm cross section became available, PTW “Pinpoint” Model 31006. This chamber was used in this study to measure transverse profiles of the clinical gamma knife beams and compare them with the film results.

## METHODS

### A. Chamber

**Figure 1:**
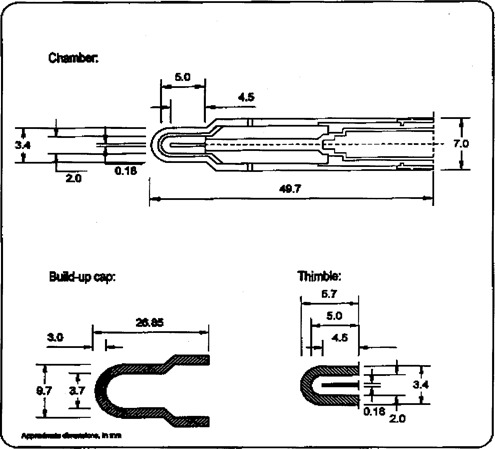
Cross‐sectional dimensions of the PTW PinPoint ion chamber.

Figure 1 shows the cross‐section of the PTW Pinpoint ion chamber. The dimensions of the chamber are 2‐mm diameter by 5 mm in length. Table I compares the dimension of the Pinpoint to the Capintec PR‐05T chamber used for calibration with the 18‐mm helmet. The PinPoint chamber has a volume of 0.015 cm.^3^ A plastic phantom, 16 cm in diameter, is supplied by Elekta and has acrylic cassette inserts for placing film, TLD, and ion chambers in the gamma knife beam. For these measurements, a blank cassette was custom drilled to accommodate the PTW Pinpoint chamber and place it at the center of the plastic phantom. The position of the chamber in the cassette was verified by film.^4^

**Table I:** Compares the dimensions of the PTW 31006 PinPoint chamber to the Capintec PR‐05T chamber used for calibration with the 18‐mm helmet.

Chamber	Length	Width (diameter)	Volume, cc
PR05P^a^	5.5 mm	4.0 mm	0.07
PTW 31006	5.5 mm	2.0	0.015

a Capintec.

### B. Mounting the chamber in the gamma knife

**Figure 2:**
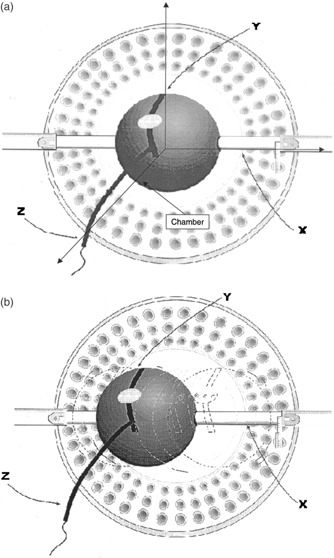
Shows the plastic phantom mounted in a gamma knife helmet on the *x*‐direction trunnions and the gamma knife coordinate system. (b) For the measurement of beam profiles, the phantom with the chamber was moved along the *x* direction using the *x*‐axis verniers to give an accuracy of 0.5 mm in position.

Prior to a gamma knife treatment, a stereotactic frame is attached to the patient's skull. The *y* and *z* coordinates for the fiducial space are set on the stereotactic frame prior to treatment. For treatment the frame, with the patient attached, is mounted on a helmet through *x*‐direction trunnions, which are set to fix the frame in the *x* direction. (Figure 2a) shows the gamma knife coordinate system and illustrates the experimental setup with the plastic phantom mounted on a helmet on the *x*‐direction trunnions. The *z* axis is oriented along the table and/or patient axis, the *x* axis is to the patient's left, and the *y*‐axis points anterior. The *x*‐direction trunnions have a 0.1‐mm vernier scale that allows accurate setting of the *x* coordinate. The estimated accuracy of the *x*‐coordinate setting is to within 0.5 mm.

*Orientation of the chamber*. For measurements of the beam profiles the plastic phantom was mounted in the helmet using the *x*‐direction trunnions. The phantom can be mounted to orient the chamber cassette in three orthogonal planes along the *x, y,* and *z* axis. For these measurements, the chamber was oriented in a sagittal plane and adjusted with the gamma anglea to orient the chamber along the *z* axis, perpendicular to the *x* and *y* direction. The sensitive volume of the chamber was in a transverse, *x,y* section of the beam.

### C. Ion chamber measurement of profiles

For the measurement of the transverse beam profiles, the phantom with the chamber was moved along the *x* direction using the *x*‐axis verniers to give an accuracy of ±0.25mm in position. See (Fig. 2b). Measurements of output were taken at a position, the phantom moved, and the measurement repeated at appropriate intervals along the *x* axis in the *xy* plane. The output values were normalized to the maximum readings.

### D. Film measurements

**Figure 3:**
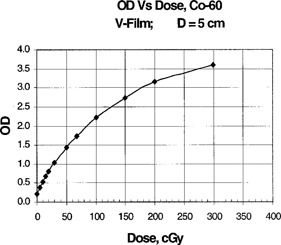
OD to dose calibration curve measured with the film at 5‐cm depth in SW using a Co‐60 teletherapy machine.

V‐film was placed in the film cassette of the 16‐cm diameter PS phantom and exposed to the clinical gamma knife beam to produce an optical density (OD) of about 2.0. The films were oriented in three orthogonal planes. The films were scanned in a Scanditronix RFA 300 film scanner with RFA300Plus version 5.2 software. The films were corrected with an OD to dose calibration curve, Fig. 3, measured with the film at 5‐cm depth in a plastic phantom using a Co‐60 teletherapy machine. Profiles were scanned through the center of the beam at 1‐mm intervals, and the beam center or maximum profile determined. The latter profile was then imported to an EXCEL spreadsheet. The ion chamber measurements were superimposed and centered upon the film profiles.

Note that the two methods result in slightly different scattering conditions since in the film measurements the phantom is fixed, while the ion chamber (and phantom) is moved across the beam. As the phantom is moved laterally [*x*], the depth of the measuring point in the phantom decreases on one side, but increases on the opposite side. The effect on the delivered dose will average out and is less than 1%. The maximum lateral phantom movement was 3 cm for the 18‐mm helmet, or 5‐cm depth on one side and 11‐cm depth on the opposite side.

## RESULTS

**Figure 4:**
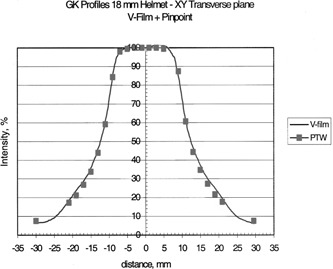
Transverse beam profiles for the 4‐mm helmet; measured with film and the PTW PinPoint ion chamber.

**Figure 5:**
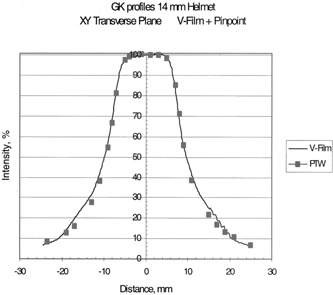
Transverse beam profiles for the 8‐mm helmet; measured with film and the PTW PinPoint ion chamber.

**Figure 6:**
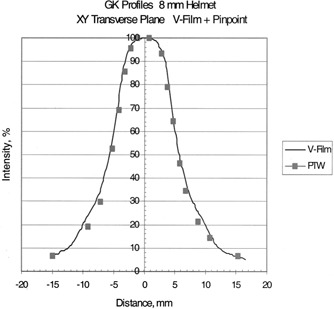
Transverse beam profiles for the 14‐mm helmet; measured with film and the PTW PinPoint ion chamber.

**Figure 7:**
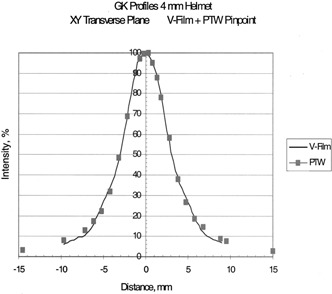
Transverse beam profiles for the 18‐mm helmet; measured with film and the PTW PinPoint ion chamber.

Table II shows the clinical beam widths at the 50% isodose lines and the penumbra widths as measured with film for the four helmets. Beam profiles of the transverse plane measured by the two methods are shown in Figs. 4, 5, 6, and 7 for the 18, 14, and 8% 4‐mm helmets, respectively. Good agreement between the film and chamber profiles is observed. The two methods agree except at the edges of the profiles, where the film results are about 1–2% higher than the chamber readings. The edge of the profiles show the “tail” of the isodose distribution that can contribute dose to adjacent structures. The 18‐, 14‐, and 8‐mm helmets show a flat 100% plateau. The 4‐mm helmet is sharply peaked and does not show any flat plateau.

**Table II:** Beam widths and penumbras in *x* direction in the transverse plane for the four helmets measured with film and the PinPoint ion chamber. Beam width at 50% isodose line, penumbra from 20–82% isodose line.

	XY plane V film		
Helmet	X, mm	Y, mm	Penumbra^a^
18 mm	20.9	23.0	11.0
14 mm	17.9	17.9	9.8
8 mm	10.9	10.9	6.2
4 mm	6.0	6.0	4.1

a Distance to decrease from 80 to 20 percent.

## DISCUSSION

The transverse profiles as measured by film show very good agreement with the ion chamber measurements. The transverse profiles show a region of flat, 100% dose for the 18‐, 14‐, and 8‐mm helmets, while the 4‐mm helmet is sharply peaked with no plateau. The beams fall off sharply as shown by the measured penumbras, Table II. The tail of the dose profiles extends to points far from the central axis. The 5% isodose extends to 3 cm from the beam center for the 18‐mm helmet, 2.5‐cm for the 14 mm, 1.5 cm for the 8 mm, and 1 cm for the 4‐mm helmet. It is important to know the beam profile shape when delivering high doses with the accuracy of the gamma knife. The isodose in the tail is 3–5% but the dose value can be appreciable for the high doses delivered in gamma knife treatments with multiple “shots.” (Five percent of 20 Gy to 50%=2 Gy). The “tails” indicate the possible interaction with adjacent treatment sites.

## CONCLUSION

These chamber measurements of transverse beam profiles verify the accuracy of the film measurements with flat isodoses for the 18‐, 14‐, and 8‐mm helmets, and a spiked isodose for the 4‐mm helmet. The dose distribution falls off rapidly, but the “tails” extend a distance from the central axis.
